# Divergent Roles of Zebrafish IGF1 Receptor a and b in Glucose and Lipid Metabolism

**DOI:** 10.3390/ijms27115013

**Published:** 2026-06-01

**Authors:** Jiankang Bao, Xing Chen, Gang Zhai, Xia Jin, Jiangyan He, Zhan Yin, Qiyong Lou

**Affiliations:** 1 State Key Laboratory of Breeding Biotechnology and Sustainable Aquaculture, Institute of Hydrobiology, Chinese Academy of Sciences, Wuhan 430072, China; 2 College of Advanced Agricultural Sciences, University of Chinese Academy of Sciences, Beijing 100049, China; 3 Fisheries College, Ocean University of China, Qingdao 266001, China

**Keywords:** IGF1 receptor, *igf1ra*/*b*, hyperglycemia, hepatic steatosis, AKT, mTOR

## Abstract

Insulin-like growth factor 1 (IGF-1) signaling plays a complementary role to insulin signaling in glucose metabolism homeostasis. This study characterized the physiological roles of the IGF1 receptor A (Igf1ra) and B (Igf1rb) in zebrafish. The transcripts of igf1ra and igf1rb were detected in multiple zebrafish tissues, including the liver, muscle, and brain. Zebrafish lacking igf1ra or igf1rb were generated using CRISPR/Cas9 technology. Both *igf1ra^−/−^* and *igf1rb^−/−^* zebrafish exhibited stunted growth. Reduced BMI was found in *igf1ra^−/−^* zebrafish, while BMI increased in *igf1rb^−/−^* zebrafish. Hyperglycemia and increased hepatic glycogen were observed in *igf1ra^−/−^* zebrafish, while blood glucose levels in *igf1rb^−/−^* zebrafish were normal. No significant difference in whole-body or hepatic triglyceride content was observed in *igf1ra^−/−^* zebrafish, while the whole-body and hepatic triglyceride content of *igf1rb^−/−^* zebrafish increased compared to their wild-type control siblings. Further analyses of the expression patterns of key genes involved in glucose and lipid metabolism were conducted on igf1r mutants. Decreased levels of genes involved in glucose absorption and glycolysis and increased levels of genes involved in gluconeogenesis and glycogen synthesis were observed in *igf1ra^−/−^* zebrafish, but not in *igf1rb^−/−^* zebrafish. Conversely, significantly decreased levels of transcripts involved in lipolysis and increased levels of transcripts involved in the lipogenesis process were observed in *igf1rb^−/−^* zebrafish, but not in *igf1ra^−/−^* zebrafish. Restricted cell growth and protein synthesis signaling, including AKT and mTOR activation, was also detected in *igf1ra^−/−^* zebrafish, while a moderate elevation in AKT and mTOR activity was seen in *igf1rb^−/−^* zebrafish. Taken together, our results suggest that functional divergence occurred after the duplication of the zebrafish igf1r gene, with igf1ra primarily modulating glucose absorption and utilization, and igf1rb primarily affecting lipid metabolism in the somatotropic axis.

## 1. Introduction

The main nutritional elements of animals are carbohydrates, proteins and lipids. Carbohydrates are utilized in the form of glucose, which can be absorbed by cells via glucose transporters. Maintaining blood glucose levels is vital for animals. Hyperglycemia and hypoglycemia can cause clinical symptoms and are harmful to the efficient utilization of nutrition. Insulin-like growth factor 1 (IGF1) is an insulin-like peptide discovered in the late 1950s. IGF1 was reported as a downstream effector of growth hormone (GH) signaling in vertebrates, thereby performing a somatotropic axis in mammals and teleost fish [1,2,3].

IGF-1 signaling was termed “somatomedin”. This emphasizes the fact that IGF1 signaling modulates body growth and the development of somatic organs. The IGF1 signaling pathway includes the IGF1 receptors [3], IRS-1 [4] and PI3K-Akt [5]. Most of the downstream effectors are cross-activated with insulin signaling. The insulin receptor substrates are also activated by IGF1 signaling [4]. IRS activation then leads to the activation of downstream pathways, PI3K and AKT/PKB, which control the actions of the somatotropic axis in regulating gene expression and the activation of enzymes involved in metabolism and development [6]. Due to genome duplication in teleost fish, an increased number of paralogous genes compared with other vertebrates has produced potential functional divergence [7,8]. The IGF1R is a class II receptor tyrosine kinase (RTK) belonging to the insulin receptor family. It plays a critical role in cell growth and differentiation, and can be activated by IGF1, IGF2, and insulin [9]. IGF1R and the insulin receptor (IR) are closely related and share 57% sequence identity, as well as displaying high structural similarity in mice [10]. Poor survival in mouse models was caused by deficient functions of Igf1, resulting in severe developmental defects [1]. However, Igf1 depletion only caused moderate defects, except for dysfunctions in glucose absorption and protein anabolism, in zebrafish [3]. Conversely, depletion of insulin signaling in mice resulted in only slight growth retardation at birth [11]. Meanwhile, much more severe defects were detected in insulin signaling-deficient zebrafish, which do not survive beyond 16 dpf [12]. This suggests divergent functions of IGF1 in mammals and teleost fish. Therefore, elucidating the regulatory mechanisms of the somatotropic axis, including GH, insulin and IGF1 signaling, is valuable for understanding nutrition and growth in teleosts.

Two forms of the insulin-like growth factor 1 receptor (IGF1R) have been identified in zebrafish: *igf1ra* and *igf1rb*. Dysfunction or dysregulation of IGF1R is a pathogenic basis for a wide range of human diseases, particularly growth defects, metabolic disorders, and cancer [13,14,15]. Given that zebrafish *igf1ra* and *igf1rb* exhibit high homology to human *IGF1R* [16], zebrafish serve as a valuable model for investigating diseases associated with dysregulation of IGF signaling. A previous study revealed that igf1ra and igf1rb depletion significantly reduced body length in zebrafish, and igf1rb plays a greater role in spontaneous muscle contractility and motoneuron development [17]. This suggests that *igf1ra* and *igf1rb* not only play important roles in growth but also have specific functions in zebrafish. Furthermore, *igf1rb* but not *igf1ra* is required for zebrafish primordial germ cell migration and survival [18]. The functional redundancy and divergence of these multiple IGF1Rs remain unclear and require further investigation. In this study, we generated *igf1ra^−/−^* and *igf1rb^−/−^* zebrafish and evaluated their respective phenotypes. We also evaluated the functional redundancy between these receptors. The results here clarified the divergent functions of *igf1ra* and *igf1rb* in zebrafish, deepening our understanding of metabolic regulation mechanisms in teleosts and helping to explain differences in somatotropic axis regulation between mammals and teleosts.

## 2. Results

### 2.1. Generation of igf1ra^−/−^ and igf1rb^−/−^ Zebrafish Using CRISPR/Cas9 Technique

**Figure 1:**
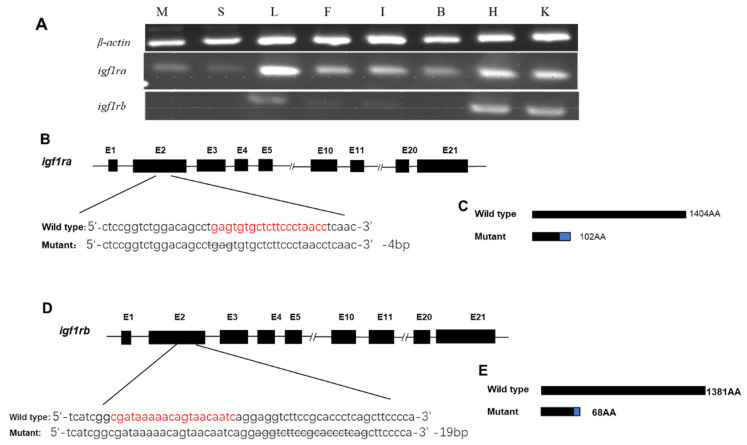
Generation of *igf1ra^−/−^* and *igf1rb^−/−^* zebrafish using CRISPR/Cas9 technique. (**A**) Detection of *igf1ra* and *igf1rb* transcripts in the tissues of adult wild-type zebrafish (M, muscle. S, spleen. L, liver. F, adipose tissue. I, intestine. B, brain. H, heart. K, kidney). (**B**) The targeting strategy of the *igf1ra* locus using the CRISPR/Cas9 technique. The CRISPR/Cas9 target is located in the exon 2. The deletion nucleotide sequence is labeled in red color. (**C**) The putative protein size of the edited premature igf1ra peptide. The black boxes and blue boxes represented the truncated wild-type sequence and the mutated peptide sequence. (**D**) The targeting strategy of the *igf1rb* locus using the CRISPR/Cas9 technique. The CRISPR/Cas9 target is located in the exon 2. The deletion nucleotide sequence is labeled in red color. (**E**) The putative protein size of the edited premature igf1rb peptide. The black boxes and blue boxes represented the truncated wild-type sequence and the mutated peptide sequence.

To investigate the regulation mechanisms of insulin/igf1 signaling, the IGF1 receptor genes were searched in the Genbank database, and the sequence of the homologs, including *igf1ra* and *igf1rb*, were searched with BLAST in the PubMed database. The protein sequence comparison results indicated that both zebrafish igf1ra and igf1rb were homologous to human IGF1R and mouse IGF1R. The expression of zebrafish *igf1ra* and *igf1rb* was detected in multiple tissues, including liver, heart and kidney. While the expression levels of zebrafish *igf1rb* were relatively lower and were detected mainly in liver, heart and kidney (Figure 1A). The CRISPR/Cas9 targets were designed using the online tool ChopChop. Both the targeting sites of *igf1ra* and *igf1rb* loci were located in the second exon, respectively (Figure 1B,D). The F0 founders of mutants were mated with wild-type zebrafish, and then the F1 offspring were genotyped. Two genetic mutant lines each were screened using Sanger sequencing for both *igf1ra* and *igf1rb* loci. Two independent lines carrying 4-bp and 59-bp deletions within the igf1ra locus, and one line carrying a 19-bp deletion within the igf1rb locus, were identified in the F1 mutants. The F1 zebrafish lines with 4-bp deletion within the *igf1ra* locus and 19-bp deletion within the *igf1rb* locus were chosen for our following studies as the *igf1ra^−/−^* and *igf1rb^−/−^* zebrafish lines, respectively (Figure 1B,D). The 4-bp deletion within the *igf1ra* locus and the 19-bp deletion within the *igf1rb* locus caused a reading frame shift in igf1ra and igf1rb peptides, respectively. The putative premature igf1ra and igf1rb peptides are shown in Figure 1C,E.

### 2.2. Growth Features and Blood Glucose Levels in igf1ra^−/−^ and igf1rb^−/−^ Zebrafish

**Figure 2:**
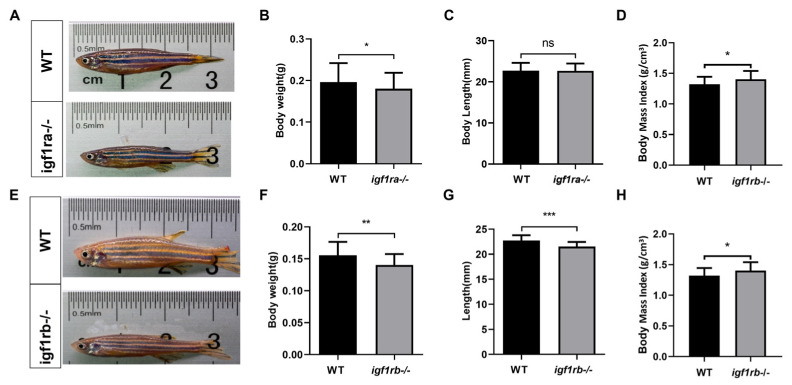
General features of the *igf1ra^−/−^* and *igf1rb^−/−^* zebrafish. (**A**) Representative appearance comparison of the *igf1ra^−/−^* zebrafish with their wild-type zebrafish. (**B**) The body weight of the *igf1ra^−/−^* zebrafish compared to that of their wild-type zebrafish (*n* = 23). (**C**) The body length of the *igf1ra^−/−^* zebrafish compared to their wild-type zebrafish (*n* = 23). (**D**) The body mass index of the *igf1ra^−/−^* zebrafish compared to that of their wild-type zebrafish. (**E**) Representative appearance comparison of the *igf1rb^−/−^* zebrafish with their wild-type zebrafish. (**F**) The body weight of the *igf1rb^−/−^* zebrafish compared to that of their wild-type zebrafish (*n* = 16). (**G**) The body length of the *igf1rb^−/−^* zebrafish compared to their wild-type zebrafish (*n* = 16). (**H**) The body mass index of the *igf1rb^−/−^* zebrafish compared to that of their wild-type zebrafish. All results are presented as mean ± standard deviation (SD) from at least three independent experiments. Differences were assessed using Student’s *t*-test. For all statistical comparisons, results were considered statistically significant at *p* < 0.05. The *p* values were recorded in the charts, and * represented *p* < 0.05, ** represented *p* < 0.01, *** represented *p* < 0.001 and ns represented no statistical significance respectively.

**Figure 3:**
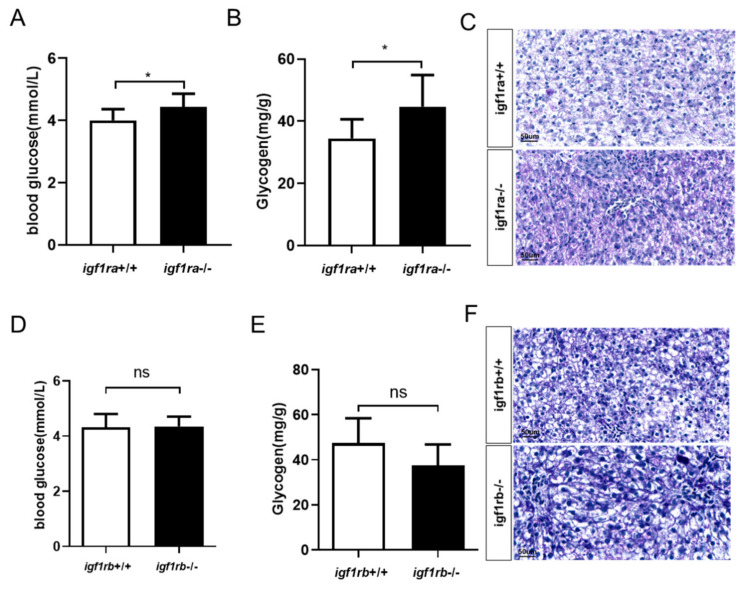
Levels of blood glucose and hepatic glycogen measurement. (**A**) Blood glucose levels in *igf1ra^−/−^* zebrafish and their wild-type zebrafish (*n* = 8). (**B**) Hepatic glycogen levels in *igf1ra^−/−^* zebrafish and their wild-type zebrafish (*n* = 6). (**C**) Representative hepatic glycogen staining of *igf1ra^−/−^* zebrafish and their wild-type zebrafish (*n* = 3). (**D**) Blood glucose levels in *igf1rb^−/−^* zebrafish and their wild-type zebrafish (*n* = 8). (**E**) Hepatic glycogen levels in *igf1rb^−/−^* zebrafish and their wild-type zebrafish (*n* = 6). (**F**) Representative hepatic glycogen staining of *igf1rb^−/−^* zebrafish and their wild-type zebrafish (*n* = 3). All results are presented as means ± standard deviation (SD) for three independent experiments. Differences were assessed using Student’s *t*-test. For all statistical comparisons, results were considered statistically significant at *p* < 0.05. The *p* values were recorded in the charts, * represented *p* < 0.05 and ns represented no statistical significance respectively.

The growth features include body length, body weight, and body mass index (BMI, weight to height ratio). The body weight of *igf1ra^−/−^* zebrafish decreased significantly, while no significant difference was observed for the body length between *igf1ra^−/−^* zebrafish and their wild-type control fish maintained in the same tank. The BMI value of *igf1ra^−/−^* zebrafish was reduced compared to the wild-type control fish (Figure 2A–D). While both the body weight and body length of *igf1rb^−/−^* zebrafish decreased significantly (Figure 2F,G), the BMI value increased compared to the wild-type control fish (Figure 2H). The blood glucose and hepatic glycogen levels of *igf1ra^−/−^* zebrafish increased compared to those of their wild-type controls (Figure 3A–C). While both the blood glucose levels and hepatic glycogen levels did not differ significantly between *igf1rb^−/−^* and wild-type zebrafish (Figure 3D–F). These diverged physical fitness test data suggested the different physiological functions between *igf1ra* and *igf1rb*.

**Figure 4:**
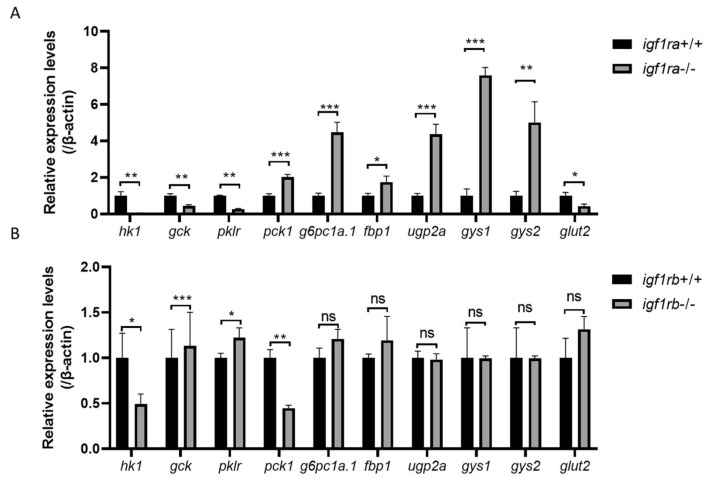
Relative expression levels of key genes involved in glucose metabolism. (**A**) Relative expression levels of key genes involved in glucose metabolism in *igf1ra^−/−^* zebrafish and their wild-type controls were maintained in the same tank, respectively. (**B**) Relative expression levels of key genes involved in glucose metabolism in *igf1rb^−/−^* zebrafish and their wild-type controls were maintained in the same tank, respectively. All results are presented as means ± standard deviation (SD) from at least three independent experiments. Differences were assessed using Student’s *t*-test. For all statistical comparisons, results were considered statistically significant at *p* < 0.05. The *p* values were recorded in the charts, and * represented *p* < 0.05, ** represented *p* < 0.01, and *** represented *p* < 0.001 and ns represented no statistical significance respectively.

The expression levels of the key genes involved in glucose metabolism were determined further. Comparison of the hepatic tissue samples *igf1ra^−/−^* zebrafish and those of their wild-type control fish, the transcriptional levels of the genes responsible for glycolysis, including *gck1*, *hk1* and *pklr* were down-regulated in *igf1ra^−/−^* zebrafish. The expression levels of genes involved in glycogen synthesis, such as *fbp1*, *ugp2a*, *gys1* and *gys2*, were upregulated in *igf1ra^−/−^* zebrafish. The glucose transporter protein coding gene *glut2* was also suppressed in *igf1ra^−/−^* zebrafish (Figure 4A). However, the transcriptional levels of the genes responsible for glycolysis, including *gck1*, *hk1*, *gck* and *pklr* exhibited differential alterations in *igf1rb^−/−^* zebrafish, with transcriptional expression levels of the other genes remaining unchanged compared with those of their wild-type controls, respectively (Figure 4B).

All the tests on the glucose metabolism indicated substantial suppression of glucose absorption and utilization, although the glycogen synthesis was up-regulated in *igf1ra^−/−^* zebrafish. Conversely, the blood glucose and hepatic glycogen levels were unaffected in the *igf1rb^−/−^* zebrafish. This indicated that igf1ra, but not igf1rb, is a major signaling responsible for the regulation of glucose uptake and catabolism.

### 2.3. Different Lipids Homeostasis Phenotypes Seen Between igf1ra^−/−^ and igf1rb^−/−^ Zebrafish

**Figure 5:**
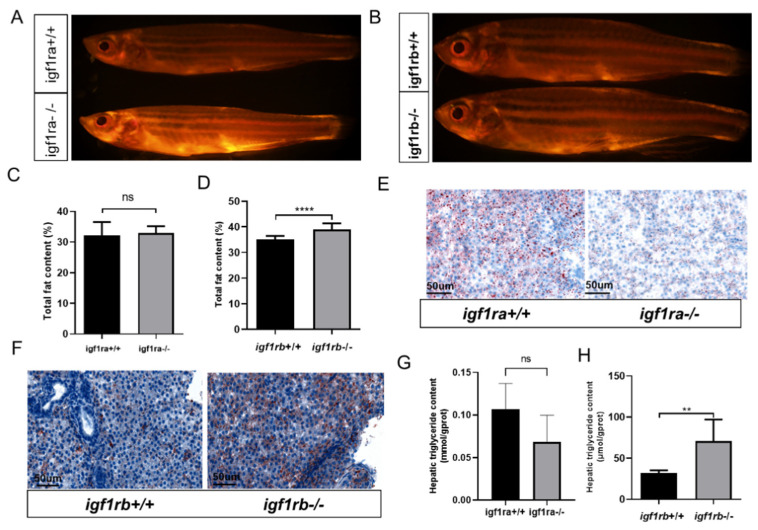
Body composition and hepatic TG content in *igf1ra^−/−^* and *igf1rb^−/−^* zebrafish. (**A**) Representative appearance and statistical analysis of whole-body fat staining of the *igf1ra^−/−^* zebrafish with their wild-type zebafish (*n* = 6). (**B**) Representative appearance and statistical analysis of whole-body fat staining of the *igf1rb^−/−^* zebrafish with their wild-type zebrafish (*n* = 6). (**C**) The whole-body fat contents of *igf1ra^−/−^* zebrafish and their wild-type zebrafish were measured using the chloroform and methanol extraction method (*n* = 12). (**D**) The whole-body fat contents of *igf1rb^−/−^* zebrafish and their wild-type zebrafish were measured using the chloroform and methanol extraction method (*n* = 12). (**E**) The oil red O staining of fat in liver sections in the *igf1ra^−/−^* zebrafish and their wild-type zebrafish (*n* = 3). (**F**) The oil red O staining of fat in liver sections in the *igf1rb^−/−^* zebrafish and their wild-type zebrafish (*n* = 3). (**G**) The hepatic triglyceride contents of *igf1ra^−/−^* zebrafish and those of their wild-type zebrafish (*n* = 6) (**H**). The hepatic triglyceride contents of *igf1rb^−/−^* zebrafish and those of their wild-type zebrafish (*n* = 6). All results are presented as means ± standard deviation (SD) from at least three independent experiments. Differences were assessed using Student’s *t*-test. For all statistical comparisons, results were considered statistically significant at *p* < 0.05. The *p* values were recorded in the charts, and ** represented *p* < 0.01, **** represented *p* < 0.0001 and ns represented no statistical significance respectively.

Since the BMI values were divergent between *igf1ra* and *igf1rb* mutants, the lipid metabolism was then explored. The whole-body fat contents were determined by Nile Red staining and chloroform/methanol extraction. There was no statistically significant difference in whole-body fat content between the *igf1ra* mutant line and their wild-type controls (Figure 5A,C,G). Although the oil red O staining data showed a decreased trend of the hepatic triglyceride levels in *igf1ra^−/−^* zebrafish compared to those of their controls (Figure 5E), this was still not statistically significantly different qualitatively (Figure 5G). Conversely, the whole-body fat content of *igf1rb^−/−^* zebrafish elevated significantly compared to that of their wild-type zebrafish (Figure 5B,D). The hepatic triglyceride content in *igf1rb^−/−^* zebrafish was also increased significantly (Figure 5F,H). These results also suggested divergent function in lipid metabolism between zebrafish igf1 receptors.

**Figure 6:**
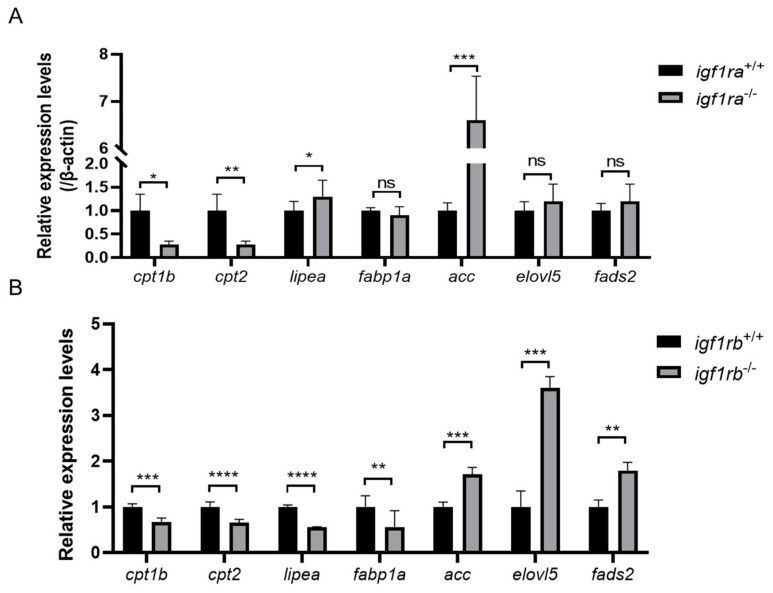
Relative expression levels of key genes involved in lipid metabolism. (**A**) Relative expression levels of key genes involved in lipid metabolism in *igf1ra^−/−^* zebrafish and their wild-type zebrafish. (**B**) Relative expression levels of key genes involved in lipid metabolism in *igf1rb^−/−^* zebrafish and their wild-type zebrafish. All results are presented as means ± standard deviation (SD) from at least three independent experiments. Differences were assessed using Student’s *t*-test. For all statistical comparisons, results were considered statistically significant at *p* < 0.05. The *p* values were recorded in the charts, and * represented *p* < 0.05, ** represented *p* < 0.01, *** represented *p* < 0.001, **** represented *p* < 0.0001 and ns represented no statistical significance respectively.

Then the activities of lipid metabolism signaling, including lipolysis and lipogenesis, were determined by analyzing the transcriptional levels of key genes. In the hepatic samples of *igf1ra^−/−^* zebrafish compared with those of their wild-type zebrafish, the transcriptional expression levels of the key genes involved in fatty acid beta oxidation, including *cpt1b* and *cpt2*, were significantly suppressed. However, the triglyceride degradation activity, which is involved in triglyceride catabolic process, and fatty acid transport activity were not affected based on the expression levels of *lipea* and *fabp1* tested. Besides, the different trends of the expression patterns of *acc*, *elovl5*, and *fads2* were seen (Figure 6A). The results suggested the integrated capacity of lipogenesis was unaffected, and this was consistent with the fat content phenotype of *igf1ra^−/−^* zebrafish seen (Figure 5A). On the other hand, the transcriptional expression levels of the gene involved in β-oxidation, triglyceride degradation, fatty acid transport, and lipogenesis were significantly repressed in *igf1rb^−/−^* zebrafish compared to those of their wild-type zebrafish (Figure 6B). Together with the BMI data and increased fat content phenotypes observed (Figure 2H and Figure 5D,H), it has been suggested that the signaling mediated by igf1rb is mainly responsible for the regulation of lipid catabolism.

### 2.4. Functional Redundancy of Insulin and Igf1 Signaling Network Coordinates Zebrafish Somatotropic Axis

**Figure 7:**
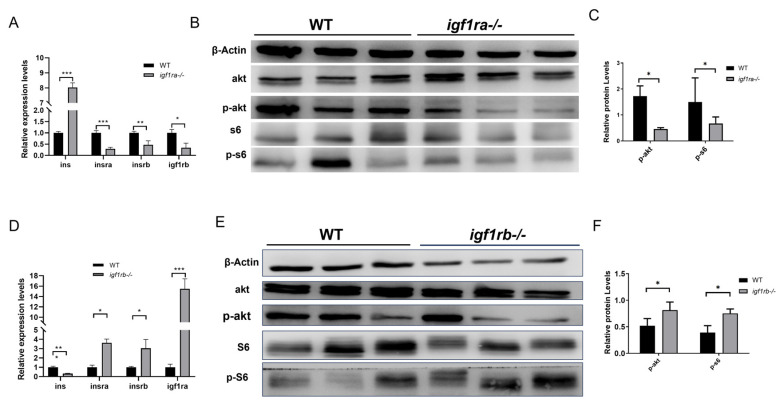
Features of some key factors involved in the somatotropic axis in *igf1ra^−/−^* and *igf1rb^−/−^* zebrafish. (**A**) Relative expression levels of *ins*, *insra*, *insrb*, and *igf1rb* transcripts in hepatic tissue samples of *igf1ra^−/−^* zebrafish with their wild-type zebrafish. (**B**) The phosphorylation statuses of AKT and S6 were examined with Western Blot analyses in the hepatic samples of *igf1ra^−/−^* zebrafish with the wild-type zebrafish. (**C**) Quantification plot of the phosphorylation statuses of AKT and S6 examined with Western Blot analyses, shown in (**B**). (**D**) Relative expression levels of *ins*, *insra*, *insrb*, and *igf1ra* transcripts in hepatic tissue samples of *igf1rb^−/−^* zebrafish compared to their wild-type zebrafish. (**E**) The phosphorylation statuses of AKT and S6 were examined with Western Blot analyses in the hepatic samples of *igf1rb^−/−^* zebrafish with the wild-type zebrafish. (**F**) Quantification plot of the phosphorylation statuses of AKT and S6 examined with Western Blot analyses, shown in (**E**). All results are presented as means ± standard deviation (SD) from at least three independent experiments. Differences were assessed using Student’s *t*-test. For all statistical comparisons, results were considered statistically significant at *p* < 0.05. The *p* values were recorded in the charts, and * represented *p* < 0.05, ** represented *p* < 0.01, and *** represented *p* < 0.001, respectively.

Following the characterization of the phenotypes of deficient zebrafish models of the two IGF1 receptors, the statuses of insulin signaling and the Akt/mTOR pathway were further explored. Firstly, the transcriptional expression levels of *insulin*, *insra*, and *insrb* were analyzed. In hepatic samples from *igf1ra^−/−^* zebrafish compared with their wild-type controls, significantly elevated *insulin* expression levels were observed, while significantly decreased *insra*, *insrb*, and *igf1rb* expression levels were observed (Figure 7A). This suggests that the insulin/IGF1 signaling pathway is attenuated due to igf1ra depletion in zebrafish. Hyperglycemia following starvation also supported Insulin signaling impairment caused by igf1ra depletion in zebrafish (Figure 3A). Western blotting analyses of the phosphorylation levels of AKT and the mTOR effector S6 in the livers of *igf1ra^−/−^* zebrafish also indicated decreased levels of phosphorylated AKT and S6 (Figure 7B,C). Conversely, the transcriptional expression levels of *insra*, *insrb*, and *igf1ra* were found to be higher in *igf1rb^−/−^* zebrafish than in their wild-type controls (Figure 7D). Consequently, the phosphorylated levels of AKT and S6 were increased in *igf1rb^−/−^* zebrafish compared to their wild-type controls (Figure 7E,F). However, unlike in *igf1ra^−/−^* zebrafish, insulin transcription was significantly downregulated in *igf1rb^−/−^* zebrafish compared to their wild-type controls (Figure 7D). This may be due to the complementary role of increased Igf1 signaling mediated by increased *igf1ra* transcript (Figure 7A), suggesting that igf1ra could complement insulin signaling in glucose metabolism.

## 3. Discussion

Blood glucose metabolism and homeostasis are among the most important aspects of growth regulation and cellular function in teleost fish [19,20,21]. Dietary carbohydrates are processed through glycolysis, glycogen turnover, and the TCA cycle, affecting growth, feed utilization, and nutrient deposition [22]. The insulin/IGF1 receptors in teleosts, such as insra, insrb, igf1ra, and igf1rb, have divergent and specific functions in metabolic modulation. In this study, *igf1ra^−/−^* and *igf1rb^−/−^* zebrafish were generated using the CRISPR/Cas9 technique. The phenotypes of the *igf1ra^−/−^* and *igf1rb^−/−^* zebrafish mutants were determined using histological and molecular methods, respectively. Compared to the Insra and Insrb ablated mutants, the overall phenotype of the igf1ra and igf1rb ablated mutants was alleviated [12,23,24]. Hyperglycemia phenotype and repressed glycolysis-related gene expression, including *gck*, *hk1*, and *pklr*, were detected in the *igf1ra^−/−^* zebrafish. This suggested that igf1ra stimulated glucose absorption and glucose catabolism. At the same time, the expression of gluconeogenesis-related catalytic enzyme genes, including *pck1* and *g6pc1a.1*, was upregulated. This confirmed that there was cellular glucose starvation in the *igf1ra^−/−^* zebrafish. It was reported that the cellular glucose starvation induced a switch in the histone acetylome for activation of gluconeogenic and fat metabolism genes [25]. However, it was different in the *igf1rb* mutants. There was no difference in the blood glucose levels between *igf1rb^−/−^* zebrafish and their wild-type controls. Glycolysis activity was stimulated moderately, and the gluconeogenesis and glucose uptake activities were unchanged in *igf1rb^−/−^* zebrafish compared to the wild-type zebrafish. The glycogen synthesis activity was also determined. The expression of *ugp2a*, *gys1*, and *gys2* elevated significantly in the *igf1ra^−/−^* zebrafish; however, there was no difference between *igf1rb^−/−^* zebrafish and their wild-type control zebrafish. The results suggest that igf1ra induces glucose uptake and glycolysis activity, which may play a significant complementary role in insulin signaling in zebrafish.

The different types of growth factors and their receptors and effectors constitute the somatotropic axis, which has redundant but unique functions. The somatotropic axis, including growth hormone (GH) [21,26], insulin (Insulin) [27], insulin-like growth factor 1 (Igf1) [3], and their receptors and effectors, has been reported to modulate lipid metabolism and triglyceride homeostasis. The total fat contents of the whole body of *igf1ra^−/−^* and *igf1rb^−/−^* zebrafish were determined. BMI data showed that the *igf1ra^−/−^* zebrafish was slimmer than the wild-type zebrafish, while there was no difference between the *igf1rb^−/−^* zebrafish and their wild-type controls. Whole-body fat and hepatic triglyceride content also indicated decreased fat deposition in *igf1ra^−/−^* zebrafish and increased deposition in *igf1rb^−/−^* zebrafish. Lipogenesis activity, as determined by the expression of key genes including *acc*, *fads2*, and *elovl5*, was increased in *igf1rb^−/−^* zebrafish. However, there were no drastic differences in the expression of lipogenesis-related genes in *igf1ra^−/−^* zebrafish, although *acc* expression was upregulated. The lipolysis cascade, as determined by the expression of key genes including *lipea*, cpt1b, and *cpt2*, was repressed in *igf1rb^−/−^* zebrafish. Therefore, the results suggested that metabolic reprogramming occurred in *igf1rb^−/−^* zebrafish. Increased glucose utilization promoted fat deposition and lipid metabolism, causing obesity. Meanwhile, glucose utilization was suppressed in *igf1ra^−/−^* zebrafish. Consequently, a slender physique and reduced hepatic triglyceride deposition were observed in *igf1ra^−/−^* zebrafish, indicating that igf1ra was responsible for glucose absorption and catabolism, while Igf1rb induced lipolysis.

Since both *igf1ra^−/−^* and *igf1rb^−/−^* zebrafish exhibited retarded somatic growth, the activation of the somatotropic axis, including insulin/IGF1, and its downstream effectors, including phosphorylated AKT and mTOR signaling, were determined. The expression of insulin/IGF1 receptors was detected, with opposite trends exhibited by *igf1ra^−/−^* and *igf1rb^−/−^* zebrafish. AKT-mTOR signaling was stimulated in *igf1rb^−/−^* zebrafish but suppressed in *igf1ra^−/−^* zebrafish. Genetic models depleted of IGF1 and insulin receptors provided an integrated evaluation of the somatotropic axis. Increased protein synthesis and decreased lipid content were reported in insulin receptor A (INSR) through stimulation of IGF1 expression [23,28]. Enhanced growth was achieved in the *insra^−/−^* zebrafish when it was fed a high-carbohydrate diet. However, growth and insulin/IGF1 signaling, including receptor expression levels and AKT-mTOR activation status, were restrained in the *igf1ra^−/−^* zebrafish. This suggests that the IGF1-IGF1Ra pathway is critical for initiating and activating the somatotropic axis, and that igf1ra is a potential and functional glucose sensor and stimulator of the somatotropic axis. It was also verified that there was a moderate improvement in glucose utilization and elevated protein synthesis in *igf1rb^−/−^* zebrafish through the stimulation of *insra*, *insrb*, and *igf1rb* expression. Although carbohydrates remain a contentious area, as many fish species are traditionally considered glucose intolerant [13], supplementing the diet with carbohydrates improves the growth of aquaculture fish species, including tilapia and largemouth bass [23,24,29,30].

Through the assay on the depletion models of *igf1ra* and *igf1rb*, divergent roles in glucose homeostasis and lipolysis of igf1ra and igf1rb were revealed in zebrafish. *igf1ra* transmits the signal of somatotropic growth factors to modulate glucose absorption and metabolism, which may complement insulin signaling. Our results provided evidence that loss of igf1ra function suppressed growth and protein synthesis signaling. Feedback upregulation of igf1ra stimulated protein synthesis signaling. This supports the idea that increased carbohydrate utilization improves the growth of teleosts. The functions of lipolysis and lipogenesis were also verified for the role of *igf1rb* in this study. As a consequence, genetic depletion of *igf1rb* caused hepatic steatosis, which counteracted the improved mTOR activity. Furthermore, the Insulin/IGF1 receptor has been shown to have substantial functional redundancy. There are complex and precise regulation optimizes metabolic homeostasis in the body.

We propose that, following whole-genome duplication, igf1ra and igf1rb have undergone subfunctionalization, with igf1rb playing a more prominent role in glucose homeostasis and igf1ra showing greater involvement in lipid metabolism during early development. This functional divergence is consistent with the subfunctionalization model, in which duplicated genes partition ancestral functions to avoid the deleterious consequences of gene loss. Given the well-established role of IGF1 signaling in human metabolic disorders—such as insulin resistance, type 2 diabetes, and non-alcoholic fatty liver disease—our zebrafish knockout models provide a valuable platform for understanding how IGF1R dysfunction contributes to metabolic dysregulation. These insights may inform future therapeutic strategies targeting the IGF1R pathway.

## 4. Materials and Methods

### 4.1. Zebrafish Strains Maintenance

Zebrafish were maintained in a circulating water system with a 14-h light and 10-h dark cycle at 28 °C. The animals were fed twice daily with newly hatched brine shrimp. To collect fertilized eggs, sexually mature male and female pairs were transferred into the tanks before the end of the light period. After the beginning of the next light cycle, the partition was removed to allow contact for laying eggs and fertilization. Embryos were then collected and stored in water containing 0.006% ocean salt. The larvae containing wildtype and homozygous were maintained and raised in the same tank. Adult zebrafish at 90 days post fertilization (dpf) were used for the assays conducted in this study following genotyping. The body weight data were collected using a precision electronic balance, followed by wiping dry with gauze. All animal experiments were conducted in accordance with the Guiding Principles for the Care and Use of Laboratory Animals and were approved by the Institute of Hydrobiology, Chinese Academy of Sciences (Approval ID: IHB 2013724).

### 4.2. Genetic Ablation of Genes Through CRISPR/Cas9

The CRISPR/Cas9 technique was used for gene editing in zebrafish according to the method described by Mali [31]. The DNA sequence of igf1ra and igf1rb was obtained from NCBI (http://www.ncbi.nlm.nih.gov/, accessed on 8 July 2023), and Guide RNAs were designed using an online software tool [32]. The knockout target site of igf1ra is (5′-TCAGGTCCTTCCCCAAGTTGACCATGG-3′), and the knockout target site of igf1rb primer sequence is (5′-GGAAAACTGTACTGTGATTG-3′). Then, using the pMD19T-gRNA backbone plasmid as template, amplified the included PCR products, introduced a T7 promoter, and the gDNA sequence. Followed the PCR products using gel extraction kit purification (Item No. D2500-01, E.Z.N.A.^®^ Gel Extraction Kit, omega, Norcross, GA, USA). Following purification using a gel extraction kit, the PCR products were used for gRNA synthesis using the TranscriptAid T7 High Yield (Item No. K0441, Fermentas, Waltham, MA, USA). Purify and measure the concentration of gRNA using a multi-functional microplate reader (Infinite^®^ M Nano, TECAN, Männedorf, Switzerland) and dilute to 1000 ng gRNA mixed with 1 μL NLS-Cas9 Nuclease and 1 μL 10× Reaction Buffer (Item No. E365-01A, NOVOPROTEIN, Wuhan, China) for microinjection immediately into zebrafish fertilized eggs at a 1–2-cell stage. For genetyping, according to the primer design tool provided by NCBI, the forward primer (5′-CCTGGACAAACATCTCCGCA-3′) and reverse primer 5′-ACACTTTCGCACACTGTCCT-3′ were designed to amplify a 324 bp PCR product covering the target site of igf1ra, and the forward primer (5′-GCGCATGTTAGAAAGTCCCC-3′) and reverse primer 5′-CGCAGATTGTAGAGGCCGAT-3′ were designed to amplify a 598 bp PCR product covering the target site of igf1ra. The PCR products amplified from the genomic DNA of fish were run in a PAGE gel after denaturation and annealing. The PAGE gel was stained with ethidium bromide for 10 min before UVB imaging. The homoduplexes and heteroduplexes were separated, with heteroduplexes being slower in mobility, and using Sanger sequencing to check for disordered peaks around the target site.

#### 4.2.1. Morphological and Phenotypic Analyses

After genotyping, body weight and body length were measured in male zebrafish. The BMI of igf1ra and igf1rb was calculated according to the method described by Frank [33]. We analyzed 23 wild-type and 23 mutant male zebrafish individuals with igf1ra knockout and analyzed 16 wild-type and 16 mutant male zebrafish individuals with igf1rb knockout.

#### 4.2.2. Nile Red Staining

Igf1ra/b and wild-type male zebrafish were immersed in pre-prepared 1 μg/mL Nile red dye. (Item No. N121291, Aladdin, Shanghai, China) for 12 h under dark conditions. Then, images were captured using an Olympus SZX16 microscope (Olympus Corporation, Tokyo, Japan) and the accompanying Olympus cellSens Entry software Ver.3.2.

#### 4.2.3. Sections Staining and Histological Analysis

Oil red O staining. The 90-day-old zebrafish hepatic tissue was sampled and placed in fixative fluid (Item No. N121291 G1119, Servicebio, Wuhan, China). Then, samples were frozen, sectioned, and stained with oil red to detect fat deposits in the hepatic samples [27]. For the detection of glycogen, the sections were stained with the PAS staining kit. The images were acquired using a stereomicroscope (Zeiss SteREO Discovery. V20, ZEISS, Oberkochen, Germany). Six samples each from wild-type and mutant zebrafish.

#### 4.2.4. Body Fat Content Assay (Chloroform and Methanol Extraction)

The contents of fish fat were assayed as per previously described procedures [27]. The adult zebrafish were anesthetized and frozen at −80 °C for 24 h (*n* = 12), then dried with a freeze-dryer (Alpha 2-4 LSC, Marin Christ, Osterode, Germany). After weighing the dry weight, 6 mL of a mixture of chloroform and methanol (volume 2:1) was added to the samples. Then, the samples were ground and sealed overnight at 4 °C to allow the fat to separate fully from the tissue. Afterwards, 2 mL of KCl (0.37 M) was added. The samples were then oscillated for 5 min. After the samples achieved stratification, they were centrifuged at 500× *g* for 20 min. Finally, the liquid in the lower layer was transferred using a Pasteur Pipette into a newly weighed tube. The extraction was first air-dried under nitrogen, and then oven-dried at 55 °C. The sample left was the total fat to weigh. Ratios of the weight of total fat/dry weight were calculated as the body fat content.

#### 4.2.5. Blood Glucose, Hepatic Glycogen Measurement

Blood samples for blood glucose measurement were collected from fasted animals. The OneTouch UltraVue (LifeScan, Devault, PA, USA) blood-glucose tester was used for the measurement of blood glucose levels, according to the manufacturer’s instructions. The hepatic tissues were collected from fasted zebrafish and washed in cold PBS buffer (pH: 7.4). The tissues were snap-frozen in liquid nitrogen until the sample test. The samples were prepared according to the manufacturer’s procedure. The Glycogen assay kits (colorimetric) were purchased from (Item No. 700480, Cayman Chemical, Ann Arbor, MI, USA).

#### 4.2.6. RNA Extraction and Relative Expression Analysis of Genes Using Real-Time PCR

Total RNA was extracted from the liver of adult zebrafish, total RNA was extracted using the RNeasy Mini kit (Item No. DP420, TransGen, Beijing, China). MMLV reverse transcriptase (Item No. 28025013, Thermo, Waltham, MA, USA) was used for reverse transcription. Primers used in the study are listed in Table 1. RT-qPCR was conducted using TransStart^®^ Tip Green qPCR SuperMix (Item No. AQ141-04, Transgen, Beijing, China) with the Bio-Rad (CFX96 Touch) software 3.1 and calculated by the Bio-Rad CFX Manager (Bio-Rad Laboratories, Hercules, CA, USA). The quantitative PCR (qPCR) program consisted of an initial denaturation step at 96 °C for 2 min, followed by 45 cycles of denaturation at 98 °C for 10 s, annealing at 60 °C for 10 s, and extension at 68 °C for 30 s. The results are presented according to the method described by Livak [34], and Technical triplicates were run for each sample. The beta-actin gene was used for internal reference in the SemiQuantitative Real-time PCR, and the fold-change of the target gene should be similar.

### 4.3. Western Blotting Analyses

Western blot analysis was performed following the methods described previously. Briefly, zebrafish hepatic samples were lysed in RIPA buffer containing proteases and phosphatase inhibitors. Total protein in samples was separated by gel SDSPAGE and transferred onto the Protran Nitrocellulose Transfer membrane (Immobilon^®^-P). The membranes were blocked for 2 h in TBS/0.1% Tween 20/5% milk. The primary antibodies used were: beta-Actin (Santa Cruz, SC-69879, Dallas, TX, USA), S6 Ribosomal Protein (Cell Signaling, BST, Danvers, MA, USA, 2217), p-S6 (Cell Signaling, BST, USA, 2215), Akt (Cell Signaling, BST, USA, 9272), p-Akt (Cell Signaling, BST, USA, 4060). Secondary antibodies used were: Peroxidase-conjugated Affinipure Goat AntiMouse IgG (H+L) (ProteinTech, Wuhan, China, A00001-1), Peroxidase-conjugated Affinipure Goat Anti-Rabbit IgG (H+L) (ProteinTech, Wuhan, China, SA00001-2), Peroxidase-conjugated Affinipure Donkey Anti-Goat IgG (H+L) (ProteinTech, Wuhan, China, SA00001-3). The signal was detected using a CCD camera-based imager (ImageQuant LAS 4000 mini, Cytiva, Uppsala, Sweden). The intensity of the bands was quantified using the ImageJ software version 1.49V.

### 4.4. Statistical Analysis

All data for RT-qPCR and western blotting intensity, analyzed by the Image J software, were expressed as the mean ± SD. The Student’s *t*-test was used for determining the statistical significance, which was defined as *, *p* < 0.05; **, *p* < 0.01; ***, *p* < 0.001. Statistical calculations were performed using the GraphPad Prism Software 8.0. Each result represents the mean of at least three independent experiments.

## Figures and Tables

**Table 1:** Primers used in this study.

Application	Gene Symbol	NCBI Accession No.	Forward Primers	Reverse Primers	Products Size (bp)
qRT-PCR	*igf1ra*	NM_152968.1	CCCGTGTTCACCTACCCA	TTGCCTTTGATGACTGTGC	207
	*Igf1rb*	NM_152969.1	GATGCGTCGCATGTGTGACAA	CAGTCAGTGATCCTGTCTGGC	214
	*igf1*	XM_073945515.1	ACTGGTGCTGTGCGTCCTC	GGTCCATATCCTGTCGGTTT	144
	*ins*	NM_131056.1	GGTCGTGTCCAGTGTAAGCA	GGAAGGAAACCCAGAAGGGG	147
	*insra*	XM_005171260.6	ATGTGCAGGACCTACGTGTG	CCATTCGTCCGGCTCATACA	203
	*insrb*	NM_001123229.1	GCTGGACGGCGGAAACTATT	CATGCGCAGGATCTCAGACT	151
	*hk1*	NM_213252.1	ACTTTGGGTGCAATCCTGAC	AGACGACGCACTGTTTTGTG	137
	*gck*	NM_001045385.2	TGAGGATGAAGAGCGAGGC	AGAGAAGGTGAATCCCAGCG	178
	*pklr*	XM_068213868.2	GCAGCAGGTGGACATGATCT	AGGATCTGCTCAAAATTGCGG	150
	*pck1*	NM_214751.1	GTGAACTGAACCGAGACCTG	AGCACTTGAGAGCAAACGAT	192
	*g6pc1a.1*	XR_012387541.1	GCTGCACCATACGAGATGGA	TCACCAAACAGCACCCACTT	248
	*fbp1*	NM_199942.4	AAGCCCCAAAGGAAAGCTCA	ATCAGGGGATCCCATCACCA	160
	*ugp2a*	XR_012401103.1	TCTTCATGTCCTGCTGTTGGG	TGTCCACTGTTGTACAGTTCC	172
	*gys1*	NM_201180.2	GGGATACACACCAGCCGAGT	GCGACTCTGCTGACAGAACT	202
	*gys2*	NM_001018679.1	TCAATGGACGCAACGACAGA	ATGACTGTGCACTCACCTGG	169
	*glut2*	NM_001042721.1	CACGAGGCCGTTTGCTG	TGCCGCTCAATGACCTTCTG	150
	*cpt1b*	NM_001328192.1	TGAGACGGATTCTTTCCGCT	TTCGCTAGGCTTGTTACTTGC	242
	*cpt2*	XM_068222837.2	AATGGATTGGGTGCAACGTG	TGAGTTCTAACCTTCAGGCTC	240
	*lipea*	XM_005159495.5	GGACCGCAGCTTACTTTTGG	TCCGACTGGATGTGAAAGTGT	191
	*fabp1a*	NM_199942.2	TCGTTCTGCTTGAACATGCG	GCTGTCTCACCATGAAGCAAC	162
	*acc*	NM_001271308.1	ATGGCAGAGCAAGACTCCAC	CCTCTGCAGGTCGATACGTC	294
	*elovl5*	NM_200453.3	TTTCGGCTAGAAGGAAAGCAG	GAACCGAAAGTGGGAGGTG	201
	*fads2*	NM_131645.2	CCAATCAGAGCGAGCCTTCA	ACGCATTCAAAGTGCCACAA	136
	*β-actin*	XM_035614479.1	ACTCAGGATGCGGAAACTGG	AGGGCAAAGTGGTAAACGCT	119

## Data Availability

The original contributions presented in this study are included in the article. Further inquiries can be directed to the corresponding authors.
